# Study on a Bayes evaluation of the working ability of petroleum workers in the Karamay region, Xinjiang, China

**DOI:** 10.3389/fpsyg.2022.1011137

**Published:** 2022-10-10

**Authors:** Hengqing An, Lei Xu, Yuanyuan Liu, Dongsheng Ma, Dajun Zhang, Ning Tao

**Affiliations:** ^1^The First Affiliated Hospital, Xinjiang Medical University, Ürümqi, China; ^2^Public Health and Preventive Medicine Post-Doctoral Mobile Station, Xinjiang Medical University, Ürümqi, China; ^3^Xinjiang Clinical Research Center of Urogenital Diseases, Ürümqi, China; ^4^College of Public Health, Xinjiang Medical University, Ürümqi, China; ^5^Department of Sanitary Technology, Department of Public Health, Karamay Campus, Xinjiang Medical University, Karamay, China; ^6^Secondary Supply Room, Disinfection Distribution Center of the First Affiliated Hospital of Xinjiang Medical University, Ürümqi, China; ^7^Karamay Central Hospital Health Management Center, Karamay, China; ^8^Department of Epidemiology and Health Statistics, College of Public Health, Xinjiang Medical University, Ürümqi, China; ^9^Clinical Post-Doctoral Mobile Stations, Xinjiang Medical University, Ürümqi, China

**Keywords:** Bayes evaluation, working ability, petroleum workers, stress, occupation

## Abstract

**Objectives:**

Use Bayes statistical methods to analyze the factors related to the working ability of petroleum workers in China and establish a predictive model for prediction so as to provide a reference for improving the working ability of petroleum workers.

**Materials and methods:**

The data come from the health questionnaire database of petroleum workers in the Karamay region, Xinjiang, China. The database contains the results of a health questionnaire survey conducted with 4,259 petroleum workers. We established an unsupervised Bayesian network, using Node-Force to analyze the dependencies between influencing factors, and established a supervised Bayesian network, using mutual information analysis methods (MI) to influence factors of oil workers’ work ability. We used the Bayesian target interpretation tree model to observe changes in the probability distribution of work ability classification under different conditions of important influencing factors. In addition, we established the Tree Augmented Naïve Bayes (TAN) prediction model to improve work ability, make predictions, and conduct an evaluation.

**Results:**

(1) The unsupervised Bayesian network shows that there is a direct relationship between shoulder and neck musculoskeletal diseases, anxiety, working age, and work ability, (2) The supervised Bayesian network shows that anxiety, depression, shoulder and neck musculoskeletal diseases (Musculoskeletal Disorders, MSDs), low back musculoskeletal disorders (Musculoskeletal Disorders, MSDs), working years, age, occupational stress, and hypertension are relatively important factors that affect work ability. Other factors have a relative impact on work ability but are less important.

**Conclusion:**

Anxiety, depression, shoulder and neck MSDs, waist and back MSDs, and length of service are important influencing factors of work ability. The Tree Augmented Naïve Bayes prediction model has general performance in predicting workers’ work ability, and the Bayesian model needs to be deepened in subsequent research and a more appropriate forecasting method should be chosen.

## Introduction

Work ability is a multifactorial concept, including workers’ health status, physical capacity, and psychosocial resources (Oellingrath et al., 2019). Work ability reflects the balanced relationship between work needs and productivity. Maintaining and improving the work ability of workers has a positive impact on promoting labor productivity and socioeconomic development. A decrease in work ability and an increase in absenteeism due to illness, decrease in work efficiency, and early retirement are increasing (Ng and Chan, 2018), and this loss of work ability will increase personal and socioeconomic burdens. Research evidence from the United States shows that the annual economic loss caused by loss of work ability is as high as $260 billion each year (Mitchell and Bates, 2011).

There are many factors affecting work ability (van den Berg et al., 2008). Some previous studies have shown that work ability is closely related to mental and psychological factors (Ruitenburgm et al., 2012). The research results of Gharibi et al. (2016) showed that the low work ability of Iranian workers was significantly related to work pressure. A research report by Sun, X, and others pointed out that the work ability of copper-nickel miners in Xinjiang decreased with the increase of job burnout; thus, reducing job burnout can improve the work ability of copper and nickel miners (Sun et al., 2020). There are also some research results showing that anxiety is closely related to work ability. Anxiety in the workplace is a long-term chronic mental health problem, which can lead to long-term sick leave and frequent absenteeism, resulting in loss of work ability (Muschalla, 2017). In a randomized controlled experimental study, the reduction of depressive symptoms could improve the work ability of workers (Hange et al., 2017). Some sociodemographic characteristics are also related to work ability. At present, the world is facing the problem of an aging labor force. The individual resources of elderly workers decrease with age, especially physical strength and certain cognitive abilities, while job needs remain the same or even increase. This leads to a reduction in work ability (Ilmarinen and Ilmarinen, 2015). In addition, sociodemographic characteristics, such as education level, marital status, obesity, and smoking, are also important factors influencing work performance (Qiaojun et al., 2017; Garzaro et al., 2019). At present, the research on the work ability of the professional population mainly involves teachers (Zuorong et al., 2015), medical staff (Xue, 2014), and manual workers (Yanxia et al., 2015). Among the manual workers in some special occupations, occupation-related characteristics play an important role in their health and work ability. For example, oil workers have unique work conditions. Their long-term shifts, irregular work and rest schedules, heavy workload, and long-term improper working postures cause long-term pain in the neck, shoulders, and lower back. Therefore, the muscles of petroleum workers, the morbidity rate of skeletal injuries (shoulder, neck, and lower back) are very high (Ning, 2016). Ge et al.’s study showed that the prevalence of musculoskeletal diseases among oil workers is as high as 84.7%, which not only affects their health but also causes long-term damage to their work ability (Ge et al., 2018). In addition, most of the workplaces of petroleum workers are in the Gobi Desert, so workers are extremely prone to mental and psychological problems, such as occupational stress, job burnout, anxiety, etc. These factors not only damage workers’ mental health but are also important factors that lead to reduced work ability (Brešić et al., 2007; Lee et al., 2017; Xiaoming et al., 2017; Xue, 2020).

The health of the occupational population has attracted much attention, and occupational health-related surveys and monitoring have gradually increased. In the process of occupational health surveying and monitoring, a large amount of information and data have been generated, but these data have not been well-exploited (Abad et al., 2019). Gerassis et al. (2018) believe that the application of new technological solutions based on artificial intelligence (AI) to occupational health databases has become a new strategy. Using big data to evaluate the results of occupational health surveys and monitoring can provide new models and ideas and explore the relationships between pieces of information to identify potential occupational health risk factors. Bayesian network is a data mining (or data-based machine learning) method that visualizes the relationship between variables as an interactive network. The result for each relationship is based on statistical conditional probability. All variables are represented by nodes. At each node, the probability distribution of the variable is defined by its relationship with the parent node, which is the root node from which all incoming vectors originate. Variables can directly or indirectly affect the outcome through other variables (Smith et al., 2018). The Bayesian network is widely used in the prediction of occupational accidents and the identification of potential factors affecting medical ability (Laaksonen et al., 2010; Mirzaei Aliabadi et al., 2018; Gerassis et al., 2019), but there is no research applying the Bayesian network model to the work ability of petroleum workers. The purpose of this study is to analyze the occupational health questionnaire database of oil workers using the Bayesian network assessment method, find the potential influencing factors and strong evidence of petroleum workers’ work ability, and establish a work ability prediction model through machine learning in order to formulate intervention measures and promote the work ability of oil workers while providing references.

## Materials and methods

### Data collection

The data comes from the 2015 oil worker health questionnaire database created in the Karamay region, Xinjiang, China. There are 4,391 copies of the questionnaire. After excluding 132 questionnaires with incomplete information, there were 4,259 complete data records of the petroleum worker health questionnaire.

### Variable definitions

The variables in the health questionnaire can be divided into four categories. The first is demographic information: gender, age, ethnicity, marital status, and education level; the second is occupational characteristics: job title, income, shift status, the third category is behavioral factors: smoking, drinking, and Body Mass Index (BMI), and the fourth category is mental health and physical health: anxiety, depression, occupational stress, musculoskeletal conditions (lower back, neck, and shoulder), high blood pressure, and work ability status. In this database, occupational stress is evaluated by the Effort Reward Imbalance (ERI) questionnaire (Siegrist, 1996), and work ability is evaluated by the Work Ability Index (WAI). In the calculation of the WAI index score, work ability is divided into poor (7–27 score), good (28–36 score), and good (37–49 score) (Xiaoming et al., 2017). Musculoskeletal status is assessed using the Nordic Musculoskeletal Questionnaire (Tanaka et al., 2001), in which bone injury is defined as having symptoms of musculoskeletal injury in one or more parts within 1 year.

#### Data preprocessing

Data preprocessing and the conversion of variable forms are important steps prior to any statistical analysis. Because the Bayesian network is a non-parametric model, continuous variables need to be discredited, and all variables defined (see Appendix Table 1). For this study, missing data were deleted during preprocessing, so there is no missing data processing step.

### Bayesian statistical analysis

#### Unsupervised Bayesian network

Bayesian networks express a set of variables and conditional dependencies *via* a Directed Acyclic Graph (DAG) (Conrady and Jouffe, 2013). This research first uses a database to establish an unsupervised Bayesian network. The unsupervised learning method represents the most accurate form of knowledge because there are no restrictions on the exploration of the potential relationships between variables in the research. The purpose of the unsupervised Bayesian network model is to discover the interdependence between a large number of variables without specifying the input or output nodes. In order to obtain the influence of the factors, it is necessary to calculate the node-force. The node-force parameter is derived from the sum of the arc force, and the concept of the arc force is based on information theory relative entropy, also known as Kullback–Leibler divergence (D_KL_) (Shannon, 1948). D_KL_ is a measure of the asymmetry of the difference between two probability distributions, P and Q. In general, P represents the true distribution of the data, Q represents the theoretical distribution of the data, and D_KL_ can be expressed as:


(1) $${\text{D}}_{\text{KL}}\left(\text{P||Q}\right)={𝔼}_{X∼P}[\log\frac{P\left(x\right)}{Q\left(x\right)}]={𝔼}_{X∼P}\left[\log\text{P}\left(x\right)-logQ\left(x\right)\right]$$

#### Supervised Bayesian network

This study established a supervised Bayesian network with work ability as the target variable. The purpose was to measure the importance of each factor to the target variable. In the supervised Bayesian network we first set the target variable according to the data and research purpose. In this research, the target variable is work ability. In order to minimize the complexity of the model, we use the Naïve Bayes algorithm to construct the supervised Bayesian network, so that mutual information is easier to calculate. Mutual information is the basic concept of information theory; it represents the amount of information held by each variable itself. Mutual information is defined as the marginal entropy of the target variable and the conditional entropy of a given target. The difference between the two variables is formally called mutual information (MI) (Conrady and Jouffe, 2015). Generally, the mutual information between two variables, X and Y, can be expressed with the following formula:


(2) M⁢I⁢(X,Y)=H⁢(X)-H⁢(X|Y)

(3) $$MI\left(X,\;Y\right)=\sum_{x\in X}\sum_{y\in Y}p\left(x,\;y\right)log2\frac{p\left(x,y\right)}{p\left(x\right)p\left(y\right)}$$

The MI value between work ability and each factor can determine which factors provide us with the greatest information gain; that is, the importance of each factor to work ability.

#### Tree augmented naïve Bayes prediction model

The disadvantage of the Naïve Bayes model is that it assumes that the features are independent of each other. However, in reality, there is no complete independence among the variables. The TAN model is a Semi-Naïve Bayes learning method (Friedman et al., 1997). It relaxes the independence assumption of the Naïve Bayes model by adopting a tree structure so that each attribute depends only on the class and another attribute. It can solve the problem of the interdependence of some attributes between conditions (Xiaolin, 2020).

The latest developments and applications of information theory have led to powerful algorithms and mechanisms, which have allowed for the optimization of Bayesian statistics in machine learning applications. According to Bayes’ theorem, Bayesian networks can provide a flexible graphical reasoning method based on the spread of uncertainty in the entire network. This study uses BayesiaLab 9.1 software to construct the Bayesian networks for probability inference and machine learning. Its artificial intelligence platform is a knowledge modeling environment that provides a variety of machine learning algorithm options, as well as an interactive environment for machine learning and knowledge discovery based on a broad set of algorithms in the Bayesian network paradigm.

## Results

### Analysis of the work ability index of petroleum workers

The average age of petroleum workers is 39.00 ± 8.60 years, and the average work ability index is 37.18 ± 5.86. Petroleum workers with low work ability account for 6.31% of the total petroleum worker population as shown in Table 1.

**TABLE 1:** Analysis of the work ability index of petroleum workers.

Work ability	Work ability index WAI${}^{\bar{x}\pm s}$	Number *n* (%)
High	41.24 ± 2.94	2,474 (58.09%)
Middle	32.82 ± 2.47	1,516 (35.60%)
Low	24.41 ± 2.78	269 (6.31%)
Total	37.18 ± 5.86	4,259 (100%)

### Unsupervised Bayesian model analyses of dependencies between variables

**FIGURE 1:**
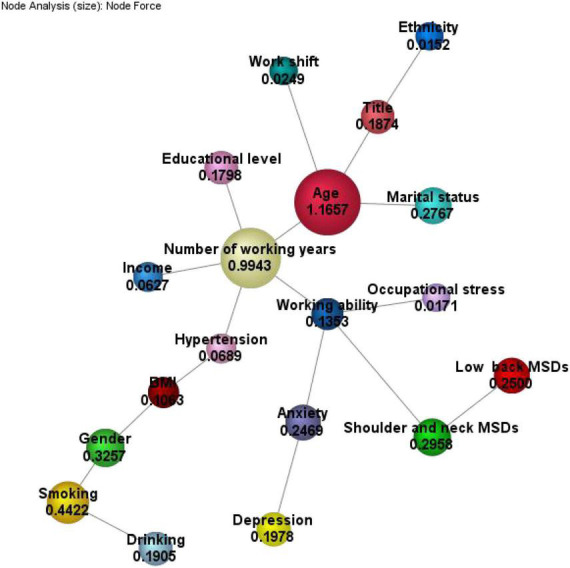
Node force analysis of unsupervised Bayesian network.

The unsupervised Bayesian model shows the relationship between variables, and the degree of dependence is proportional to the magnitude of the node-force. In this Bayesian network, we find that age, working age, and work ability are at the core of the network, as well as the starting point of other dependencies. Among them, marital status, working age, job title, and shift status are directly related to age, working age, education level, income level, working ability, and hypertension. There are four sub-nodes of work ability in the figure, namely shoulder and neck MSDs, anxiety, working age, and occupational stress, indicating that work ability is directly dependent on these four variables. The node-force shows the strength of the dependent relationship in the process of workers’ health survey. Among them, age, working age, gender, smoking, and shoulder and neck MSDs are the most influential factors in the survey process (see Figure 1).

### Sensitivity analysis of supervised Bayesian network

**FIGURE 2:**
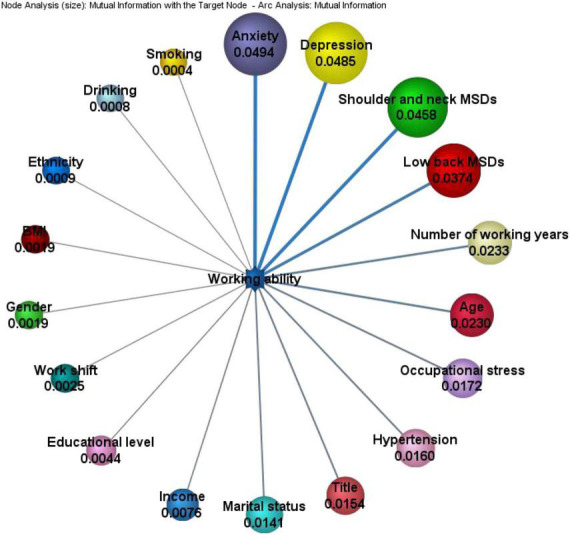
Sensitivity analysis of supervised Bayesian network. The numbers in the figure are calculated by MI. The sizes of the circles and the thickness of the lines are proportional to the importance of the influencing factors.

A sensitivity analysis can rank the importance of factors affecting work ability. The supervised Naïve Bayes model with work ability as the target node shows that the MI between computing nodes can determine which factors provide the greatest information gain for work ability. The Bayesian Network graph is sorted clockwise according to the size of the information gain. Figure 2 shows the MI between all nodes and the target node, including anxiety, depression, shoulder and neck diseases, low back MSDs, and shoulder and neck MSDs. The MI value of the factor node and work ability is relatively large; that is, these five factors are the main influencing factors that affect work ability, and the other factors have relatively little influence on work ability (see Figure 2).

### Bayesian probability interpretation tree models of work ability

**FIGURE 3:**
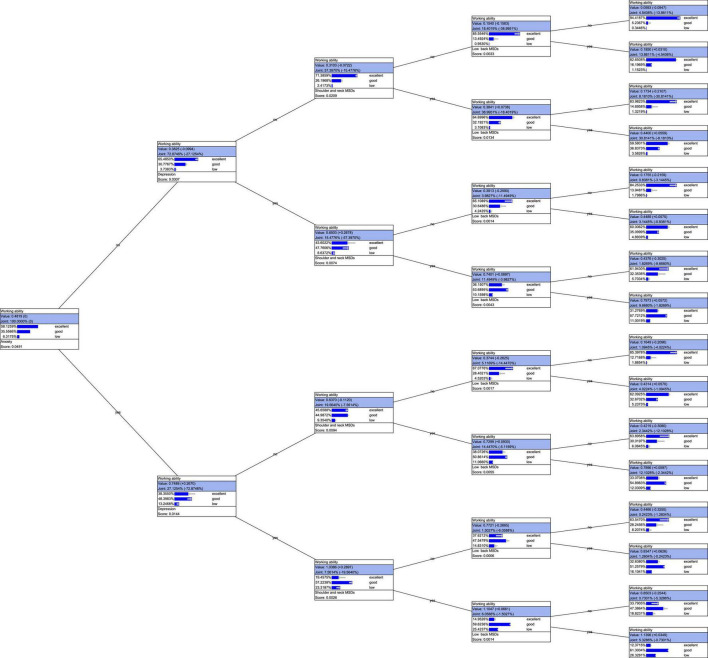
Bayesian probability interpretation tree model of work ability. In Figure 3, the arrows in the figure indicate the trends of the probability distribution changes, the joints in the tree are the joint probabilities, and the score is equal to the mutual information MI score.

The probabilistic interpretation tree model is a static tree structure that shows the influence of the different states of various factors on the probability distribution of the work ability state. The leftmost node in the tree (without any evidence set) shows the marginal probability distribution of the work ability. The probability distribution is the probability that each state of work ability occurs separately without any evidence. Considering the size of the graph, this study sets the maximum number of variables to four. The bottom panel of the leftmost node in the figure shows anxiety as the most important variable to be considered. The two branches that appear from the node represent the two MDs of the shoulder and neck. There are two different states, namely presence and absence, and we can see that when anxiety (Yes), depression (Yes), shoulder and neck MSDs (Yes), and low back MSDs (Yes) occur at the same time, the joint probability is 5.33%. At this time, the probability distribution of work ability considered high accounts for 12.37%, the probability distribution of work ability considered medium accounts for 61.16%, and that considered poor accounts for 26.46%. The joint probability of not having anxiety (No), depression (No), shoulder and neck MSDs (No), and waist and back MSDs (No) at the same time is 4.53%. The probability distribution of work ability considered high is 94.41%, the probability distribution of work ability considered medium is 5.24%, and the probability of work ability considered poor is The distribution accounts for 0.34% (see Figure 3).

### Tree augmented naïve Bayes prediction model

It can be seen in Figure 1 that there is a mutual dependence between variables;, that is, variables are not independent of each other, so we use the TAN prediction model. In the model, the work ability is set as the target variable to be predicted. Since the target variable is a multi-categorical variable, we need to convert it into a binary variable. According to the literature review by Yajia et al. (2000), comprehensive work ability is measured by the average of the scores minus one standard deviation as the cut-off value. The comprehensive work ability score is divided into two groups. The cut-off value of this study is a score of 31. Those with a score of less than 31 are considered the work ability decline group, while those with a score greater than or equal to 31 are considered the normal work ability group. Other factors are regarded as influencing variables that affect the state of the target variable. Then, we use the TAN algorithm, automatically implemented by BayesiaLab, to establish a Bayesian prediction model.

#### Comprehensive forecasting ability analysis of the tree augmented naïve Bayes forecasting model

First, we divide the data into 10 equal parts, then dividing the data into a training set and a test set at a ratio of 8:2. In order to avoid the model becoming too complicated, we use the variable selection method in the TAN model. The model finally selects occupational stress, anxiety, depression, shoulder and neck MSDs, age, and hypertension, which were modeled as characteristic variables in the model. The comprehensive evaluation of the predictive ability was made based on the accuracy, reliability, and area under the Receiver Operating Characteristic (ROC) curve, as shown in Table 2.

**TABLE 2:** Evaluation index analysis of the Bayesian forecasting model using different numbers of variables.

Data set	Accuracy (%)	Reliability (%)	AUC (%)
Training set	86.71	82.37	74.03
Test set	82.61	77.39	68.54
10-fold cross validation	86.68	82.23	72.49

## Discussion

With the growth of the world economy and the advancement of science and technology, the petroleum industry has developed rapidly and has taken a pivotal position in the world economy. The development of the petroleum industry has also made great contributions to China’s national economy. Karamay City, Xinjiang, China, is an industrialized city dominated by the petroleum industry, and its labor force accounts for 70.36% of the permanent population (Yang, 2014). Oil workers’ physical and mental health, as well as their work ability, directly affect the production safety and economic benefits of the oil industry, and it is particularly important to monitor the work ability of the professional population (El Fassi et al., 2013). Although the Karamay City Occupational Health and Hygiene Service Organization provides annual physical examinations and surveys for oil workers, the data generated by these physical examinations and surveys are difficult to make good use of. This research aims to explore the relationship between information related to work ability in health surveys by using Bayesian network assessment methods.

The Bayesian network model is helpful for revealing the complex network relationship among the factors related to work ability. The unsupervised Bayesian network used in this study shows that work ability is directly related to occupational stress, shoulder and neck musculoskeletal diseases, anxiety, and length of service. Studies have found that long-term occupational stress can cause changes in the behavior and functions of the occupational population (such as hypertension, headaches, and muscle tension), a weakened response to external stimuli, and decreased work ability and physiological functions (Mazloumi et al., 2012). Depression and anxiety are manifested as absenteeism, which leads to a decline in work ability (Muschalla, 2017). Musculoskeletal diseases are also a common cause of reduced work ability. This study shows that shoulder, neck, and lower back musculoskeletal diseases are relatively important factors influencing work ability by monitoring Bayesian networks and combining mutual information scores. The musculoskeletal system of oil workers is under high stress for a long time and cannot be relieved, which leads to neck and back pain among other musculoskeletal discomforts. These diseases cannot be treated in time so that work ability is also affected (Bugajska and Sagan, 2014; Ge et al., 2018). This study also further explained the influencing factors of work ability through the goal interpretation tree model. When there were no anxiety, depression, shoulder and neck MSDs, or low back MSDs, 94.41% of such oil workers had high work ability. Petroleum workers with poor work ability who did not have any of these conditions accounted for only 0.34%, but of the workers for whom these factors were present at the same time, 26.46% had poor work ability. This shows that these factors can explain the status of workers’ work ability to a significant extent. Anxiety and depression are important indicators of mental health. MSDs in the shoulders and neck and MSDs in the waist and back are important indicators of physical health. This also shows that the work ability of oil workers is affected by social, psychological, and physiological factors.

Working years (or natural age) is also an important factor affecting work ability. Although workers’ work experience and skills improve with the increase of working years, workers’ health and work ability gradually decline with age, especially in a job with high physical demand (Cadiz et al., 2019). It is worth mentioning that the results of this study show that the relationship between BMI and work ability is weak, which is contrary to the results reported in the literature by Andersen et al. (2017). Andersen et al. (2017) insist that obesity is associated with various diseases and functions. The decline is related to the decline. Overweight and obesity will lead to a decline in workers’ work ability. People engaged in sedentary work have a more obvious correlation between BMI and workability than those engaged in physical activities. The nature of the work of oil workers is mostly manual labor. This may be the reason for these inconsistent conclusions. In addition, this study also shows that there is no significant relationship between shifts and work ability, which is consistent with the research conclusion of Fischer et al. (2006), but there are also studies that have shown shifts and work ability being negatively correlated. Shift work may lead to burnout and work errors, which affect work ability (Elovainio et al., 2010). The relationships between BMI, shift work, and work ability need further research.

To predict the work ability of oil workers, this study established a TAN model. The accuracy of the TAN prediction model after 10-fold cross-validation was 86.68%, the reliability was 82.23%, and the AUC was 72.49%. The model has a good prediction accuracy performance, but the classification performance is average. One of the reasons for this may be that the sample imbalance causes the minority data to be predicted incorrectly or mistakenly regarded as noise and processed as such (Spelmen and Porkodi, 2018). The second reason may be that the work ability measured in this research is a multi-classification problem. Due to the imbalance of the sample, when evaluating the classification effect of the imbalanced data model, AUC is often more compatible and distinguishable than the classification accuracy (Feng et al., 2007), while AUC is suitable for evaluating two classification problems. In order to be able to use the AUC value, we divide work ability into two classification problems. This causes changes in the attributes of the sample and may affect the overall model performance. In addition, the data scale and category distribution also affect the model prediction, an important factor of ability (Xi and Kun, 2019).

The relationship between occupational stress and mental health among oilfield workers in an arid desert environment in the Xinjiang region of China was found in previous studies and revealed a causal relationship between occupational stress and psychological disorders, while the relationship between psychological disorders and genetic levels was further explored (Jiang et al., 2021). Workers in the mining, oil and gas extraction industries appear to have worse chronic health conditions than the general working population until retirement (Robinson et al., 2021), and we have found similar phenomena in our study, which deserve further attention. In Nigeria, where oil is the mainstay of the industry, almost all oil workers suffer from work-related musculoskeletal disorders (Omojunikanbi et al., 2022). It is recommended that health management training and occupational assessment programs be provided for workers in specialized industries. There is a need to develop measures to alleviate the occupational stress of oil workers in order to improve the mental health of this specific occupational group, as well as recommending ongoing attention to health status after retirement. This study uses Bayesian networks not only to visualize health management data, but also to build and validate tree-enhanced plain Bayesian models of work ability in relevant work scenarios, which reflect the correlation between the various factors in a more intuitive way. It is not limited to the occupational mental health management of oil workers, but can be further investigated in medical research, engineering information and other areas with the help of Bayesian predictive model selection and inference. This study also has certain limitations. First, the cross-sectional study cannot determine the order in which various factors and work ability occur. Second, the sample source is limited to Karamay City, Xinjiang, China, so the results may not necessarily represent oil workers nationwide. In the end, the imbalance of the sample will affect the overall performance of the prediction model. In the follow-up, we still need to conduct a more extensive investigation of the work ability of oil workers and conduct additional research on the prediction model. Details will be elaborated later. This study is reasonable and novel in applying Bayesian statistical methods to the information mining and predictive modeling tools of influencing factors related to oil workers’ work ability. The overview is reflected in the first application of Bayesian statistical methods to the study of oil workers’ work ability, as well as the information mining and visualization operations of related influencing factors and the development of predictive models, and in the methodological presentation of Bayesian statistical methods in the field of relevant public health and health research centered on oil workers.

## Conclusion

This study shows that it is reasonable to apply Bayesian statistical methods to information mining and predictive models of factors related to the work ability of oil workers. Our research results have determined that anxiety, depression, MSDs in the shoulder and neck, MSDs in the waist and back, working age, and occupational stress are all relatively important factors influencing the work ability of oil workers. This provides new insights for improving the working ability of oil workers. Through the Bayesian network, the relationship between the various factors was visualized, the Tree Augmented Naïve Bayes (TAN) model of work ability was established, and the model evaluation was carried out. The results showed that the follow-up of the Bayesian prediction model selection and reasoning still needs to be studied in-depth to provide a scientific basis for establishing a more appropriate predictive model of work ability in the future.

### The theoretical and practical implications of the study

The health of occupational groups is of great concern, and relevant questionnaires and monitoring for characteristic occupational groups are gradually increasing, while a large amount of information and data are generated in the process of occupational health surveys and monitoring, but these data have not been well-exploited. The aim of this study is to analyze the occupational health data of oil workers in the Karamay region of China through a Bayesian network evaluation method, to identify potential influencing factors and strong evidence of oil workers’ work capacity, and to build a predictive model of work capacity through machine learning, so as to provide some reference basis for government interventions and public health decisions at a later stage.

### Strengths and limitations of this study

The study uses big data to evaluate the results of occupational health surveys and monitoring, which can provide new models and ideas, and explore the interrelationships of information to identify potential occupational health risk factors.

(1) Innovatively, the Bayesian network model is applied to the work ability of oil workers for the first time. Use mutual information analysis method (MI) and Bayesian target interpretation tree model to observe the changes in the probability distribution of work ability classification under different states of important influencing factors, and establish Tree Augment Naïve Bayes (TAN) prediction Model, predict and evaluate work ability.

(2) The purpose of this study is to analyze the occupational health questionnaire database of oil workers through Bayesian network assessment, find the potential influencing factors and strong evidences of oil workers’ working ability, and establish a working ability prediction model through machine learning to formulate interventions Measures and promotes the work ability of oil workers to provide a reference basis.

(3) The study is a cross-sectional study, so it is impossible to establish a causal relationship between factors related to work ability.

(4) The study is conducted in the oil-operating population of Karamay, a resource-based city in Northwest China, and due to special occupations This may affect the extrapolation of research conclusions.

## Data availability statement

The original contributions presented in this study are included in the article/supplementary material, further inquiries can be directed to the corresponding authors.

## Ethics statement

Written informed consent was obtained from the individual(s) for the publication of any potentially identifiable images or data included in this article. This study was reviewed by the Ethics Committee of Xinjiang Medical University (No. 2015006) and approved on June 26, 2015. Written informed consent to participate in this study was provided by the patient/participants’ or patient/participants legal guardian/next of kin.

## Author contributions

NT and DZ designed this study. LX and YL collected and managed these data. DM completed the data analysis. HA and LX drafted the manuscript. NT checked and revised the manuscript. All authors have read and approved the final manuscript.

## Appendix

**APPENDIX TABLE 1:** Definition of discrete variables.

Variable	Definitions
Age	<30 years old, 30–44 years old, ≥45 years old
Gender	Male, female
Shift status	Fixed day shift, two shifts, others
BMI	<18.5 kg/m^2^ means underweight, 18.5–24.9 kg/m^2^ means normal weight, 25–29.9 kg/m^2^ means overweight, ≥30 kg/m^2^ means obesity
Ethnicity	Han and ethnic minorities
Marital status	Unmarried, married, divorced, widowed
Job title	None, elementary, intermediate, deputy senior, and senior
Income	Per capita monthly income is low < 3,000 yuan, monthly per capita income is 3,000–4,500 yuan, monthly per capita income is high > 4,500 yuan
Drinking history	No drinking, drinking
Smoking history	No smoking, smoking
Education level	Secondary vocational school or college or below, university and above
Occupational stress	Yes, no
Anxiety	Yes, no
Depression	Yes, no
Hypertension	Yes, no
MSDs of back	Yes, no
MSDs of neck and shoulders	Yes, no
Working experience	<10 years, 10–25 years, more than 25 years
Work ability	High, medium, low
